# Modelling the impact of COVID-19 and routine MenACWY vaccination on meningococcal carriage and disease in the UK

**DOI:** 10.1017/S0950268823000870

**Published:** 2023-06-01

**Authors:** Liza Hadley, Andromachi Karachaliou Prasinou, Hannah Christensen, Mary Ramsay, Caroline Trotter

**Affiliations:** 1 Disease Dynamics Unit, University of Cambridge, Cambridge, UK; 2 Population Health Sciences, University of Bristol, Bristol, UK; 3 UK Health Security Agency, London, UK

**Keywords:** COVID-19, meningococcal, modelling, social distancing, vaccine uptake

## Abstract

Country-wide social distancing and suspension of non-emergency medical care due to the COVID-19 pandemic will undoubtedly have affected public health in multiple ways. While non-pharmaceutical interventions are expected to reduce the transmission of several infectious diseases, severe disruptions to healthcare systems have hampered diagnosis, treatment, and routine vaccination. We examined the effect of this disruption on meningococcal disease and vaccination in the UK. By adapting an existing mathematical model for meningococcal carriage, we addressed the following questions: What is the predicted impact of the existing MenACWY adolescent vaccination programme? What effect might social distancing and reduced vaccine uptake both have on future epidemiology? Will catch-up vaccination campaigns be necessary? Our model indicated that the MenACWY vaccine programme was generating substantial indirect protection and suppressing transmission by 2020. COVID-19 social distancing is expected to have accelerated this decline, causing significant long-lasting reductions in both carriage prevalence of meningococcal A/C/W/Y strains and incidence of invasive meningococcal disease. In all scenarios modelled, pandemic social mixing effects outweighed potential reductions in vaccine uptake, causing an overall decline in carriage prevalence from 2020 for at least 5 years. Model outputs show strong consistency with recently published case data for England.

## Introduction

Enforced country-wide social distancing and suspension of almost all non-emergency medical care due to the COVID-19 pandemic will undoubtedly affect health and healthcare in a wide variety of ways. Public health interventions designed to interrupt the transmission of the SARS-CoV-2 virus will have effects on other infectious diseases, particularly those spread through respiratory secretions. In addition, routine healthcare, including the national immunisation programme, has been disrupted by the pandemic. This paper estimates both pre-pandemic and pandemic carriage prevalence, examining the expected effect of this disruption and COVID-19 interventions on meningococcal disease in the UK, with a specific focus on the adolescent MenACWY vaccine.

Meningococcal disease, caused by the bacterium *Neisseria meningitidis* can result in meningitis and septicaemia, both of which are serious and life-threatening. One in 20 cases of meningococcal disease results in death in the UK, and one in five survivors will have a permanent disability (skin scars, limb amputation, hearing loss, seizures, or brain damage). The five most prevalent types of the bacterium are routinely vaccinated against in the UK, using the Hib/MenC, MenB, and MenACWY vaccines, given during infancy and adolescence [1]. It is also possible to be a ‘carrier’ of *N. meningitidis.* ‘Carriage’ refers to an asymptomatic infection where the bacteria colonise the mucosa of the nasopharynx but do not cause invasive disease. In comparison to disease, carriage is relatively common, and it is possible for individuals to have multiple carriage episodes [2].

First, we estimated pre-pandemic carriage prevalence and then explored the potential impact of the pandemic on this disease. Understanding the effect of COVID-19 on meningococcal disease is not a trivial problem. The potential future increase in transmission of meningococci from reduced MenACWY vaccine uptake (due to COVID-19 distancing, isolation, and reduced healthcare capacity) may or may not outweigh the potential decrease due to extensive country-wide social distancing. These two competing factors must be studied in detail. Mathematical models of transmission are a useful tool for examining a range of scenarios.

We adapted an existing mathematical model for meningococcal carriage, to assess the following:

- What is the predicted impact of adolescent MenACWY vaccination on pre-pandemic meningococcal transmission?
- What effect might social distancing and reduced vaccine uptake both have on the future epidemiology of meningococcal carriage and disease?
- Will catch-up vaccination campaigns be necessary for the MenACWY vaccine?

## Methods

### Model structure

**Figure 1.**
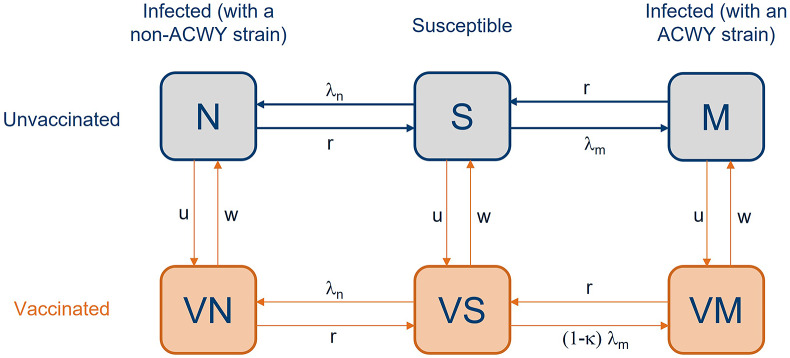
The 6-compartment SIS model for carriage used in this paper, adapted from [3]. Individuals are either susceptible (S), carriers with a vaccine-preventable meningococcal strain (M), or carriers with a non-vaccine-preventable meningococcal strain (N). Rows separate individuals that are unvaccinated from those who are vaccinated and protected (V). Arrows dictate the allowed movement between compartments, where λ_m_ and λ_n_ represent the force of infection for vaccine-preventable and non-vaccine-preventable meningococcal strains respectively, κ denotes vaccine efficacy against carriage acquisition, and *r* denotes rate of recovery (1/duration of carriage). u denotes vaccine uptake and 1/w denotes waning vaccine protection. Cases are not represented in the model diagram but occur on acquisition of carriage.

We adapted an existing published model for meningococcal carriage, which was originally designed to assess the potential impact of the MenB vaccine. Full model details are given in [3] and in the Supplementary Material. This is an age-structured compartmental transmission-dynamic model, where individuals were characterised as either susceptible to infection (S) or infected. For infected individuals, one distinguishes between those infected with a vaccine-preventable meningococcal strain (denoted M) and those infected with a non-vaccine-preventable strain (denoted N). The S, M, and N compartments are shown as columns in Figure 1 and were divided based on whether the individual is unvaccinated or vaccinated with the MenACWY vaccine. The vaccine was assumed to provide a degree of protection against ACWY carriage and disease, although cases of the disease were computed outside of the model using an age-specific case:carrier ratio and were not explicitly modelled (Supplementary Table S2). Vaccine protection could wane, in which event, individuals in compartment VS returned to compartment S. Infected individuals who recovered could also subsequently become reinfected and progress through the model in an analogous manner. Seasonality was not included in the model.

To convert to modelling the MenACWY vaccine, the adapted model assumed that vaccine-preventable strains were meningococcal groups A, C, W, and Y only, with annual routine vaccination beginning in 2015. All other serogroups (including meningococcal serogroup B) were treated as non-vaccine-preventable. Parameter values were updated to account for these changes and are shown in Table 1. The equilibrium (pre-2015) carriage prevalence curve was also scaled uniformly by 40% to account for the lower prevalence observed in teenagers by UKMenCar4, a 2014 UK carriage prevalence survey [4]. This survey would have captured the effect of previous UK meningococcal vaccination programmes, such as the mature MenC programme [7]. Further details of model fitting, scaling, and validation are given in the Supplementary Material.

**Table 1.** Model parameters

Parameter name		Notation	Value	References
Proportion of meningococcal strains protected through vaccination		p.vac	32%	[4]
Routine vaccine uptake (2015 catch-up campaign)	13 years	u	85%	[5]
	14–18 years	u	70%	[5]
	18–25 years	u	35%	[5]
Routine vaccine uptake (from 2016)	13 years	u	85%	[5]
Vaccine efficacy against carriage		κ	41%	Estimated from fitting to [5]
Duration of carriage		1/r	6 months	[3]
Vaccine efficacy against disease		d	94%	[6]
Vaccine waning (time after vaccination)		1/w	10 years	[3]

κ sensitivity analysis is plotted in Supplementary Figure S3.

An observational study of meningococcal carriage prevalence before and after the start of the MenACWY vaccination programme was used to estimate κ, the vaccine efficacy against carriage due to vaccine groups [5]. Carr et al. [5] observed a 65% decrease in carriage prevalence from 2015 to 2018. Fitting our model to these study results produced a vaccine efficacy against carriage best estimate of 41% for ACWY strains [97.5% CI: 38–44] with 0% for all other strains.

It is important to note that the model was not designed as an elimination model and does not account for stochastic die-out or reintroduction. The model does not also account for processes such as novel strain introduction, which are known to occur [8].

### Pandemic social mixing assumptions

Most transmission of *N. meningitidis* occurs through close contact with an asymptomatic carrier [9]. By understanding how frequently members of a population typically interact with each other, one can quantify the likely number of opportunities for infection spread.

These social interactions are captured in a social contact matrix. The original Christensen et al. [10] social contact matrix used assortative mixing, where individuals are assumed more likely to interact with others of their own age, one year older, or one year younger, compared to the rest of the population. This was motivated by a study conducted in an earlier work by Trotter et al. [11], where a reported 80% of secondary meningococcal cases from 1995 to 1998 were within two years of that of the index case, and 57% had an age difference of less than one year [11]. We used this assortative mixing as the standard (‘pre-pandemic’ and ‘post-pandemic’) social mixing assumption.

To approximate ‘pandemic’ social mixing, a literature search was performed in June 2020 (and updated in February 2022) to identify published studies of age-stratified UK contact patterns during the COVID-19 pandemic. Searches featured combinations of keywords such as ‘social’, ‘physical’, ‘distancing’, ‘mixing’, ‘contact’, ‘age’, ‘matrix’, ‘UK’, ‘COVID-19’, and associated synonyms.

Many publications identified in the literature search focused on COVID-19 transmission. These early analyses often assumed homogeneous social mixing or assumed that social mixing occurs at pre-pandemic rates. For example, the 2006 POLYMOD survey measured the day-to-day social interactions of nearly 8,000 participants in 8 European countries and is widely referenced [12].

Two novel studies of age-stratified pandemic mixing were identified: the UK CoMix study and a multi-country study by Del Fava et al. [13] and [14]. Surveys from CoMix captured the mean number of daily social contacts between and within age groups of a UK study population, with initial estimates being made for under 18’s using the 2006 POLYMOD dataset as a baseline [14]. Overall, data from March to October 2020 indicated an approximated 75% reduction in daily social contacts for periods of school closures and an approximated 60% reduction for periods of school openings. Del Fava et al. [13] observed similar changes. These values represent an average across all age groups; age-stratified daily social contact data was only available for the first week of the survey at the time of model construction, but large reductions have since been recorded across all age groups [15].

To derive our assumptions for pandemic social mixing, we multiplied our standard social contact matrix by 25% for periods of school closure and by 40% for periods of school openings. This represents the approximated 75% and 60% reduction in social contact behaviours observed by CMMID COVID-19 Working Group et al. [14] across the first six months of their study. More conservative estimates are shown in Supplementary Figure S5 and an age-stratified estimate is shown in Supplementary Figure S6.

Our model assumed that pandemic social mixing was in place for 18 months, from end of March 2020 to end of September 2021. Schools were modelled as ‘closed’ (i.e., 75% contact reduction) for April, May, June 2020 and January, February, March 2021. For all other months in the pandemic timeframe, a 60% contact reduction was used to account for the observed relaxation in COVID-19 measures and restrictions [16].

### Additional pandemic modelling assumptions

Vaccination was implemented continuously as individuals reached their 14th birthday. To explore the effects of reduced healthcare capacity, we simulated a 34% drop in regular UK MenACWY vaccine uptake for the full 18-month pandemic timeframe (March 2020 to September 2021). This value was taken from the MenACWY vaccine uptake data for the 2019/2020 school year [17]. We also carried out a sensitivity analysis on this parameter value and show an example in Supplementary Figure S4.

### Scenarios

Model scenarios for an assumed 18-month pandemic timeframe (end of March 2020 to end of September 2021) are shown in Table 2. We additionally explored a shorter pandemic timeframe of 15 months, where social distancing and reduced vaccine uptake were assumed to cease end of June 2021. This scenario was labelled ‘rapid return’ and was chosen to align with the UK government’s COVID-19 roadmap, where the majority of social distancing restrictions were removed at the end of June 2021 [16]. This was not the primary scenario as the general public remained cautious, schools shortly closed for the summer holidays, and routine healthcare continued to be delayed beyond June 2021 [18].

**Table 2.** Model scenarios for the 18-month pandemic timeframe

Model scenario	Pandemic length	Social distancing?	Reduced vaccine uptake?
	(Beginning March 2020)	(75% reduction in daily social contacts for periods of school closure; 60% reduction o/w)	(34% reduction for duration of pandemic timeframe)
No pandemic	0 months	No	No
Pandemic	18 months	Yes	Yes
Rapid return	15 months	Yes	Yes
Reduced vaccine	18 months	No	Yes
Social distancing	18 months	Yes	No

The model was run to equilibrium without vaccination for 100 years (Supplementary Figure S1), after which annual routine vaccination and one-off catch-up vaccination begin in January 2015 as per the introduction of the MenACWY vaccine to the UK [19]. Simulations then run for 5¼ years with standard vaccination and mixing (January 2015–March 2020), 1½ years with the pandemic adjustments described above (end of March 2020–end of September 2021 or end of June 2021 for ‘rapid return’), and a further 14¼ years with standard vaccination and mixing (up to December 2035). Outside of the 18-month pandemic timeframe, the standard pre-pandemic social mixing matrix and 85% vaccine uptake were assumed. Running the model to the year 2035 allowed us to observe a possible evolution of the annual routine immunisation and explore how quickly meningococcal carriage prevalence rates might recover, if at all, post-pandemic. However, the main focus of this modelling study was the short (2–5 years) impact.

## Results

### Pre-pandemic results

**Figure 2.**
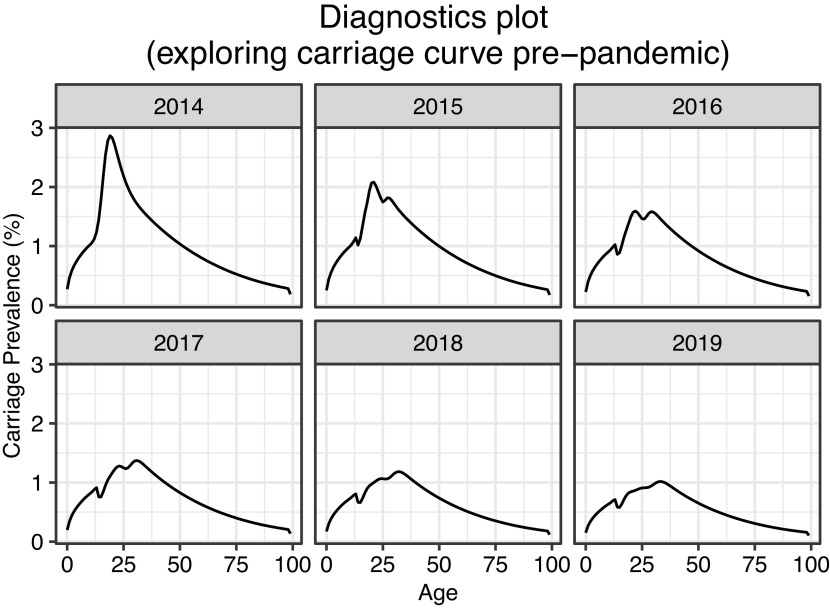
Expected early effects of UK MenACWY routine vaccination and catch-up campaigns. Vaccination was introduced in 2015. Plots depict annual UK carriage prevalence by age for vaccine-preventable (ACWY) strains.

Figure 2 demonstrates the early effects of the UK MenACWY routine vaccination and catch-up campaign. The plot depicts model outputs for the years 2014 to 2019, where ‘2014’ (top-left) indicates equilibrium carriage prevalence before the introduction of this vaccination programme. We observed a significant pre-pandemic decline in meningococcal carriage.

### Pandemic results

**Figure 3.**
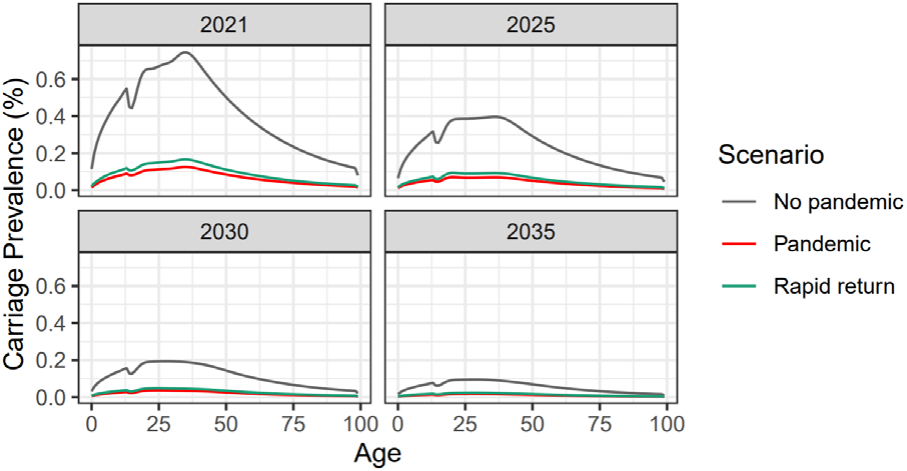
UK carriage prevalence by age for vaccine-preventable (ACWY) strains. Results are shown for year-end 2021, 2025, 2030, and 2035. Three scenarios are presented: no pandemic (grey); pandemic (red); and rapid return (green).

**Figure 4.**
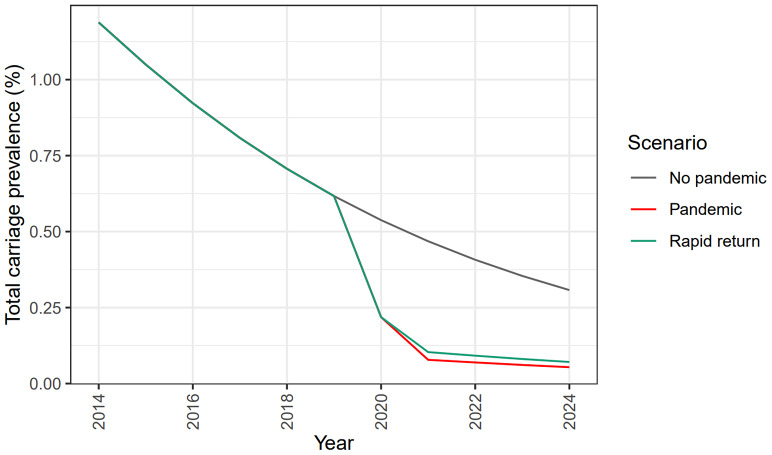
Total UK carriage prevalence over time for vaccine-preventable (ACWY) strains. Simulations are shown from 2014 for 10 years. Three scenarios are presented: no pandemic (grey); pandemic (red); and rapid return (green).

We present model outputs for our most realistic scenario – social distancing together with reduced vaccine uptake (labelled ‘pandemic’ as in Table 2). This pandemic scenario is compared against the baseline ‘no pandemic’, and a ‘rapid return’ scenario, where pandemic modelling assumptions were in place for 15 months rather than 18 (see Table 2 for the details of each model scenario). Full results for carriage prevalence are shown in Figures 3, 4 and disease estimates in Table 3. For the latter, a case:carrier ratio was derived at equilibrium with best fit to Public Health England 2015 case data. This case:carrier ratio was then multiplied by carriage incidence from model outputs to estimate future case numbers. When appropriate scalings were made to convert from a UK population to an England population, the 2015 and 2019 total case counts shown in Table 3 matched closely to published English case data [20].

**Table 3.** Prediction of case counts of UK invasive meningococcal disease for vaccine-preventable (ACWY) strains

Scenario	Year	<1	1–4	5–9	10–14	15–19	20–24	25–44	45–64	65+	Total IMD cases
No pandemic	2014	30	50	15	8	47	17	29	84	136	416
	2019	17	29	9	4	8	4	16	52	78	219
	2021	13	22	7	3	6	3	12	40	60	167
	2025	7	13	4	2	4	2	7	23	34	97
	2030	4	6	2	1	2	1	3	11	17	47
	2035	2	3	1	0	1	0	2	6	8	23
Pandemic	2014	30	50	15	8	47	17	29	84	136	416
	2019	17	29	9	4	8	4	16	52	78	219
	2021	1	2	1	0	1	0	1	4	6	18
	2025	1	2	1	0	1	0	1	4	6	17
	2030	1	1	0	0	0	0	1	2	3	9
	2035	0	1	0	0	0	0	0	1	2	4
Rapid return	2014	30	50	15	8	47	17	29	84	136	416
	2019	17	29	9	4	8	4	16	52	78	219
	2021	2	3	1	1	1	1	2	6	9	26
	2025	2	3	1	0	1	0	2	5	8	22
	2030	1	2	0	0	0	0	1	3	4	11
	2035	0	1	0	0	0	0	0	1	2	6

Results are shown by age group for the year-end 2014 (equilibrium), 2019, 2021, 2025, 2030, and 2035. Again, three scenarios are presented: no pandemic, pandemic, and rapid return.

Modelling focused on a short (2–5 year) timeframe to inform timely UK vaccination policy. Some model results are presented beyond the year 2025 for scientific interest, but it is noted that the model does not consider strain reintroduction or stochastic die-out.

In Figures 3, 4 and Table 3, we observe a great and long-lasting reduction in both meningococcal ACWY carriage prevalence and disease, initially reducing peak carriage prevalence by over 80% (from 0.75% to 0.125% in Figure 3). Figure 4 shows the expected behaviour in transmission over time. With no simulated COVID-19 outbreak, we observed a steady but significant reduction as more age cohorts received the routine MenACWY vaccine, with total carriage prevalence dropping to around 0.3% by the year 2024. In contrast, the ‘pandemic’ scenario saw a drastic sharp decline over the years 2020 and 2021, with total carriage prevalence dropping under 0.1%.

Figures 3, 4 and Table 3 also show the ‘rapid return’ scenario. Here we assumed that pandemic distancing stopped abruptly in June 2021, rather than running until September 2021. Even with this ‘rapid return’ to normality, the prevalence was substantially lower than in the no-pandemic scenario.

**Figure 5.**
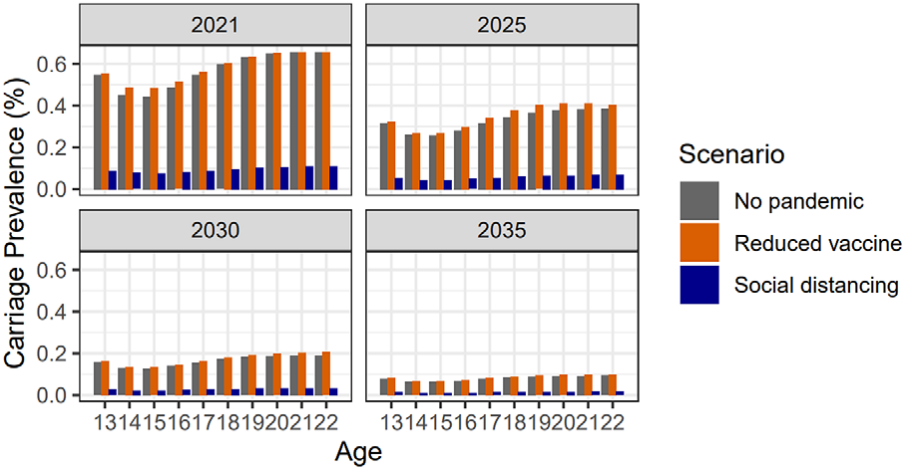
Exploring the relative weighting of pandemic effects. Plots show predicted carriage prevalence in teenagers and young adults. Results are shown by age for the years 2021, 2025, 2030, and 2035. Three scenarios are presented: no pandemic (grey); 34% reduced vaccine uptake for 18 months (orange); and social distancing for 18 months (navy). One can clearly see that social distancing outweighed reduced vaccine uptake.

In Figure 5, we explore the relative effects of social distancing versus reduced vaccine uptake using the scenarios described above. Our baseline (no-pandemic) scenario is in grey. The reduced vaccine uptake scenario is in orange, and the social distancing scenario is in navy. We observe that reduced vaccine uptake causes a very minor increase in carriage prevalence, but this is negligible in comparison to the drastic decrease from social distancing. This observation also held when a larger vaccine uptake reduction of 50% was considered (Supplementary Figure S4) and when more conservative social distancing assumptions were used (Supplementary Figure S5).

Results for non-vaccine-preventable strains are not presented in the main text as this study was primarily focused on the changing prevalence of vaccine-preventable strains. However, individuals who contracted non-vaccine-preventable strains were still included in the model and experienced reductions in pandemic social mixing in the same way as susceptible and ACWY-infected individuals (Supplementary Figure S7). Note that a constant force of infection was used for non-vaccine-preventable strains.

## Discussion

Model findings indicate that the MenACWY vaccine programme was already generating indirect protection and suppressing transmission before the pandemic began (Figure 2). This supports the findings of Carr et al. [5], who observed a 65% reduction in carriage prevalence from 2014 to 2018. Even without pandemic modelling assumptions, we observe that carriage prevalence could approach very low levels by 2035 due to the new lower community carriage prevalence observed by the UKMenCar4 study and the effects of the 2015 MenACWY catch-up campaign (Figures 3, 4).

With regards to pandemic modelling (Figures 3– 5), findings indicate that COVID-19 social distancing is expected to have accelerated the decline in carriage prevalence and disease incidence of meningococcal strains A, C, W, and Y. In all scenarios modelled, pandemic social distancing outweighed potential falls in vaccine uptake (of up to 50%). This is plausible as 18 months of country-wide social distancing affects the whole UK population, whereas 18 months of reduced vaccine uptake affects only those in the current vaccination age cohorts. Furthermore, we observed potential elimination scenarios in the long term. We would not, therefore, expect a rapid rebound in meningococcal disease, as has been predicted for other infections [21]. Results for non-ACWY strains are not presented in the main text as this study is primarily focused on the changing prevalence of vaccine-preventable strains prompted by social distancing and the disruption to the adolescent MenACWY vaccination programme through school closures. Individuals who contracted non-ACWY strains in the model still experienced reductions in pandemic social mixing in the same way as susceptible and ACWY-infected individuals. Changes in vaccine uptake of infant meningococcal vaccination programmes were not modelled, as it was assumed these would not be adversely affected by school closures. Data has since been published indicating that this is a valid assumption: for the years 2018–19 to 2021–22, uptake decreased by only 0–0.5% for primary MenB vaccination, remained steady for the MenB booster, and decreased by only 0–1.5% for the Hib/MenC booster [22].

There are a few limitations to note. Firstly, our model was not designed as an elimination model. We did not account for reintroduction but equally, we did not account for stochastic die-out. As we observe potential elimination scenarios, further assessment with a stochastic model may be helpful. The focus of this study was a short (2–5 years) time frame to determine if immediate catch-up campaigns would be necessary for the known circulating ACWY strains.

In terms of data limitations, we must note that pandemic social mixing assumptions were an average across all age groups, and an average over periods of school closures or school openings. This decision was made to make the best use of data available at the time of model construction, but additional sensitivity analysis has been carried out with an age-stratified social contact reduction shown in Supplementary Figure S6.

Second, in using the CoMix social contact survey to approximate contact behaviours, we were implicitly assuming that the definition used for direct contact in terms of possible COVID-19 exposure was also appropriate for possible meningococcal exposure. For CMMID COVID-19 Working Group et al. [14], direct contact was defined as ‘anyone who was met in person and with whom at least a few words were exchanged, or anyone with whom the participants had any sort of skin-to-skin contact’. This is a much stronger definition than that typically used to define the close contact of a person with meningococcal disease (which only includes much longer exposure periods or anyone in direct contact with a patient’s oral secretions [23]). This is an important limitation – the contact patterns incorporated into our model are hence not based on the exact types of contact that best facilitate meningococcal transmission. However, to account for this, we carried out sensitivity analysis on the parameter for reduction in daily social contacts using more conservative estimates (see Supplementary Figures S5 and S6). A significant and long-lasting decline was still observed.

Third, the age group of the 2014 UKMenCar4 survey of UK carriage prevalence was very limited, reporting only on 15–19-year-olds [4]. It was assumed that the same natural (pre-pandemic) percentage reduction seen in carriage prevalence in 15–19-year-olds occurred across all age groups. This survey was carried out in 2014 before the introduction of the adolescent MenACWY vaccine.

For disease, the case:carrier ratio (Supplementary Table 2) is a best estimate derived from data. The case:carrier ratio for 15–19-year-olds is two and half times greater than for 20–24-year-olds. Public Health England routinely observed significantly more cases in the 15-19y age category compared to the 20-24y category from 2010 to 2015, while Christensen’s systematic review observed similar carriage rates in these two age categories [24, 25]. This results in a greater estimated case:carrier ratio for the 15-19y age category. There is not a clear biological explanation for this, but there are slight differences in the carriage and case data from which the ratio is derived. For example, Christensen’s systematic review was performed in 2010 and was not serogroup specific. The review also included carriage surveys from outside the UK, but only included those countries with a similar epidemiological profile.

It is important to also note that the case:carrier ratio for invasive meningococcal disease could change in the future, perhaps for example if *SARS-CoV-2* continues to circulate widely. It is unclear if there is any association between *SARS-CoV-2* and IMD [26, 27] but an increased risk of invasive bacterial disease has been observed following other viral infections [28, 29].

With these limitations in mind, model findings are still important for informing vaccination strategy in the UK and the scenarios presented represent our best understanding of meningococcal ACWY transmission. Our model showed the MenACWY vaccine programme generating substantial indirect protection and suppressing transmission by 2020. COVID-19 social distancing is expected to have accelerated this decline, causing significant long-lasting reductions in the carriage prevalence of meningococcal strains A, C, W, and Y and the incidence of invasive meningococcal disease. Preliminary surveillance studies such as Brueggemann et al. [30] have also observed significant reductions in cases of invasive meningococcal disease in the UK in March–May 2020 (the end of the surveillance period). An overall decline in cases of IMD following the Covid-19 pandemic has also been observed in two studies performed in Italy and Australia [31, 32]. Moreover, our model estimates of disease have since been compared to recently published case data for England, showing strong consistency: our modelling estimated a total of 19 cases of invasive meningococcal disease in 2021 for serogroups A, C, W, and Y (Table 3). Official UK Health Security Agency statistics recently identified 16 cases in England across these serogroups, which when scaled to a UK population equates to approximately 18 cases [33]. Our mathematical modelling closely matches this real-world observed data, at least in the short term. It hence seems unlikely that a MenACWY catch-up campaign is necessary in the UK due to the persisting low carriage prevalence we predict.

## Data Availability

Details of the model, data inputs, and other assumptions are provided in the Methods section and Supplementary Material. Researchers interested in further model details are invited to view the online code repository at https://github.com/LizaHadley/meningolockdownmodel.

## Supplementary material

For supplementary material accompanying this paper visit https://doi.org/10.1017/S0950268823000870. click here to view supplementary material
