# Modelling the impact of COVID-19 and routine MenACWY vaccination on meningococcal carriage and disease in the UK – ERRATUM

**DOI:** 10.1017/S095026882300105X

**Published:** 2023-07-06

**Authors:** Liza Hadley, Andromachi Karachaliou Prasinou, Hannah Christensen, Mary Ramsay, Caroline Trotter

**Affiliations:** 1 Disease Dynamics Unit, University of Cambridge, Cambridge, UK; 2 Population Health Sciences, University of Bristol, Bristol, UK; 3 UK Health Security Agency, London, UK

When this article was originally published in Epidemiology & Infection it contained errors in its figures and an error in the display of a reference. Figure 2 has been updated and the sizes of figures 4 and 5 have been adjusted.

The line ‘Two novel studies of age-stratified pandemic mixing were identified: the UK CoMix study and a multi-country study by Del Fava et al. [13] and [14]’ has been updated to ‘Two novel studies of age-stratified pandemic mixing were identified: the UK CoMix study and a multi-country study by Del Fava et al. [13, 14].’

The publisher apologises for this error.
