# Supplementary material for: Modelling the impact of COVID-19 and routine MenACWY vaccination on meningococcal carriage and disease in the UK
Source: Epidemiol Infect. 2023 Jun 1;151:e98. doi: 10.1017/S0950268823000870 (PMC10284610; doi:10.1017/S0950268823000870)
Supplement: Supplementary file 1 [file S0950268823000870sup001.docx]

**Supplementary Material**

***Model equations***

The differential equations governing our meningococcal transmission model were as follows:

$$\frac{{dS}_{i}}{dt}= -\left( \lambda_{mi}+\lambda_{ni} \right)S_{i}+r\left( M_{i}+ N_{i} \right)-uS_{i}+w{VS}_{i}-mS_{i}$$

$$\frac{{dM}_{i}}{dt}= {\lambda_{mi}S}_{i}-rM_{i}-uM_{i}+w{VM}_{i}-mM_{i}$$

$$\frac{{dN}_{i}}{dt}= {\lambda_{ni}S}_{i}-rN_{i}-uN_{i}+w{VN}_{i}-mN_{i}$$

$$\frac{{dVS}_{i}}{dt}= -\left( {\left( 1- \right)\lambda}_{mi}+\lambda_{ni} \right)VS_{i}+r\left( {VM}_{i}+ VN_{i} \right)+uS_{i}+w{VS}_{i}-m{VS}_{i}$$

$$\frac{{dVM}_{i}}{dt}= {\left( 1- \right)\lambda}_{mi}VS_{i}-r{VM}_{i}+uM_{i}-w{VM}_{i}-m{VM}_{i}$$

$$\frac{{dVN}_{i}}{dt}= \lambda_{ni}VS_{i}+rVN_{i}+uN_{i}-w{VN}_{i}-m{VN}_{i}$$

This model was adapted from Christensen et al [1]. Individuals were divided into 100 single-year age classes (0 to 99 years), denoted by the subscript $i$ in the above equations. When individuals age, they move from age class $i$ to age class $i+1$. Births are introduced into the susceptible compartment $S_{0}$, and all those in age class 99 are assumed to die one year on. Movement of individuals due to infection and vaccine status is explained in Figure 1 in the main text.

***Calculating the contact matrix and force of infection***

An assortative social contact matrix was used in the model, where individuals are assumed more likely to interact with others of their own age, one year older, or one year younger, compared to the rest of the population. Methodology follows that of Christensen et al [2], reproduced here for clarity.

Preferential mixing was assumed within one year of a given age group with degree *ε*, with individuals mixing with all age groups randomly with degree 1-*ε*. For ages 0 and 99, only individuals aged 0 and 1, and 98 and 99, contribute to the preferential mixing band respectively. Epidemiological data was used to derive estimates for the initial vaccine-preventable force of infection, $\lambda_{i(0)}$, while the model was at equilibrium, and through rearrangement of the below equation, *β_i_* values were derived:


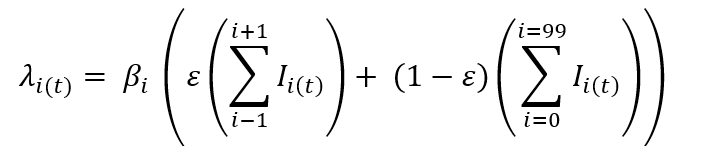
.

The $\beta_{i}$ values used in the model are shown in Supplementary Table 1.

Optimal *ε* values were calculated for a given duration of carriage by minimising the sum of squares between the prevalence of carriers in the model averaged over 1 year and that obtained from the Christensen et al. carriage systematic review [3] once the model had been allowed to stabilise (100 years). ε = 0.98 was found to be optimal. The final social contact matrix for the model was $\beta_{i}L$ where $\beta_{i}$ takes the equilibrium-derived values shown in Supplementary Table 1 and $L$ is the following matrix of $(1 - \varepsilon)$ values:


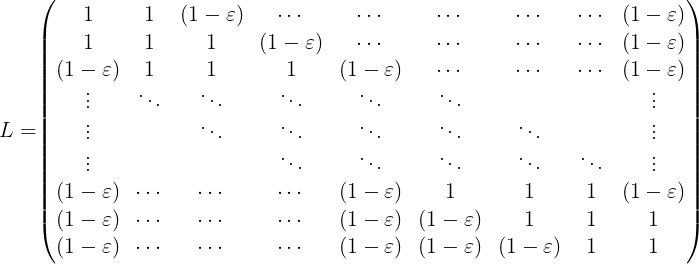


Multiplying $\beta_{i}L$ by the sum of infected individuals in each age class at time $t$ reproduces the equation for $\lambda_{i(t)}.$The time-varying force of infection $\lambda_{i(t)}$ is hence calculated within the model at each timestep, using the equilibrium-derived *β_i_* values, *ε* = 0.98, and $I_{i(t)}$ denoting the number of ACWY-infected individuals in age class $i$ at time $t$.

| **Ages**  **(years)** | ***β_i_*** |  | **Ages**  **(years)** | ***β_i_*** |
| --- | --- | --- | --- | --- |
| 0  1  2  3  4  5  6  7  8  9  10  11  12  13  14  15  16  17  18  19  20 | 0.0688  0.0782  0.0802  0.0831  0.0858  0.0882  0.0904  0.0923  0.0940  0.0954  0.0965  0.0978  0.1003  0.1056  0.1147  0.1263  0.1364  0.1415  0.1415  0.1387  0.1348 |  | 21-25  26-30  31-35  36-40  41-45  46-50  51-55  56-60  61-65  66-70  71-75  76-80  81-85  86-90  91-95  96-99 | 0.1256  0.1170  0.1120  0.1074  0.1027  0.0980  0.0934  0.0889  0.0846  0.0807  0.0773  0.0747  0.0730  0.0717  0.0688  0.0449 |

***Supplementary Table 1: β_i_ values used in the model. These were derived at equilibrium.***

***Model fitting and sensitivity analysis***

Supplementary Figure 1 compares our adapted R model to the best model fit from Christensen et al.’s systematic review [3]. The adapted model was subsequently scaled at equilibrium by 40% to account for the lower community carriage prevalence observed in the 2014 UKMenCar4 study compared with Christensen et al.’s original model and systematic review [3][4].


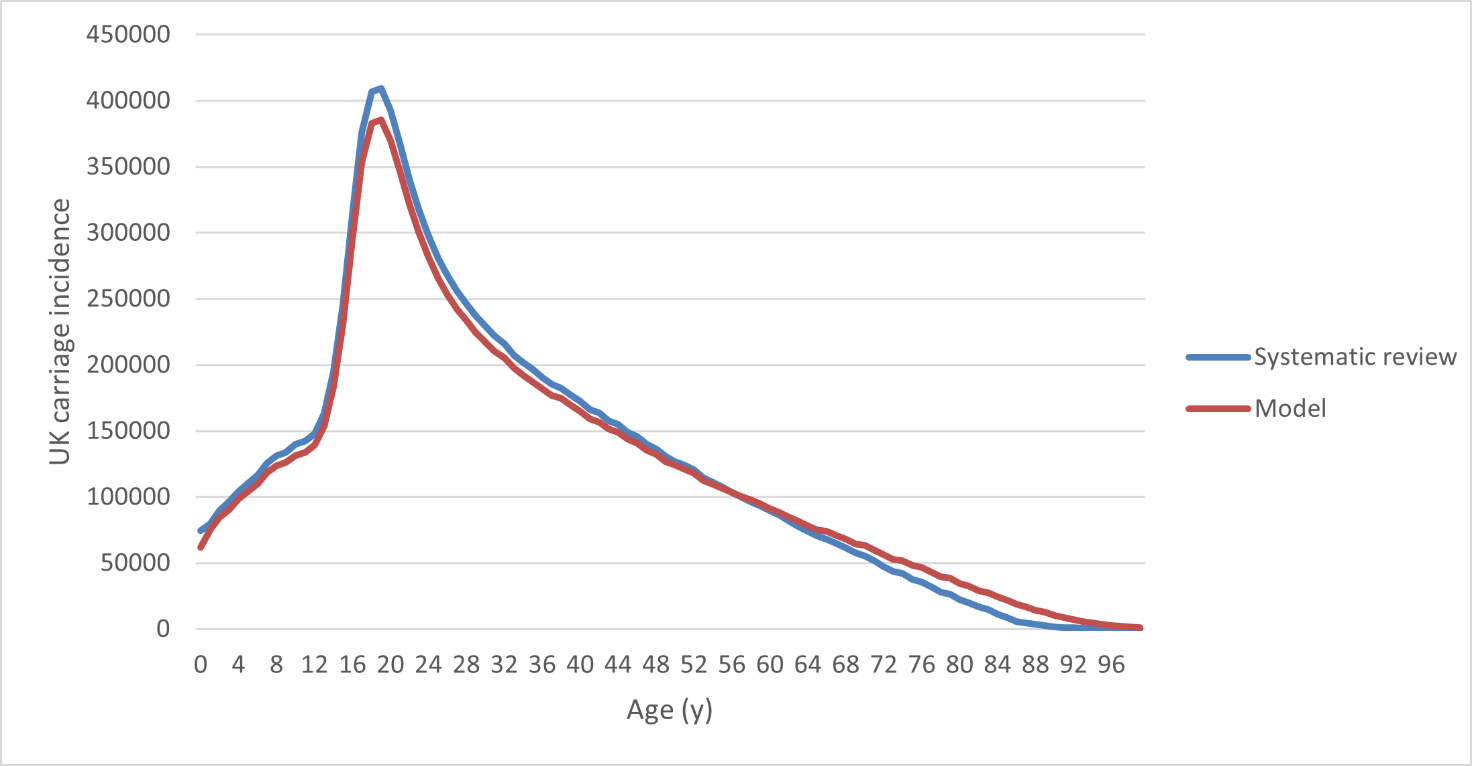


***Supplementary Figure 1: Comparison of total UK carriage incidence (all strains) of the adapted model at equilibrium (red) against the best model fit from Christensen et al.’s systematic review (blue)* [3]. *The adapted model was re-coded in programming language R and here uses the same parameter values as Christensen et al. to allow for comparison.***

Supplementary Figure 2 shows our fitting of the adapted model to recently published English datasets. Case counts were fitted to Public Health England data stratified by individual age cohorts [5]. Due to data-sharing constraints, we have only shown the fitting by broad age groups. Case fitting enabled derivation of an equilibrium case:carrier ratio for England and it was assumed that this case:carrier ratio was applicable to the whole of the UK (Supplementary Table 2).


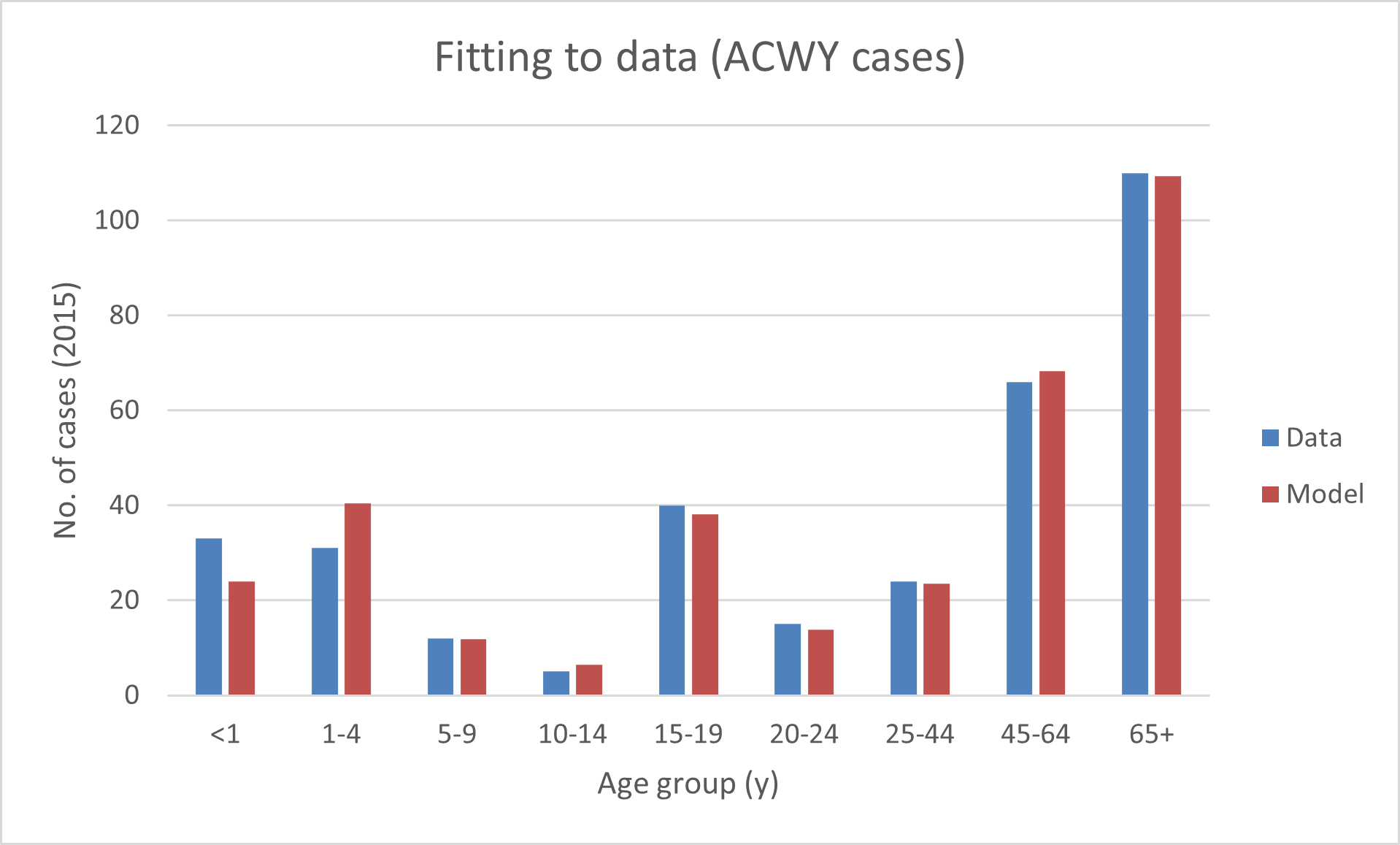


***Supplementary Figure 2: Comparison of ACWY case number predictions for England for the adapted R model at equilibrium (red) against Public Health England 2015 data for cases of Invasive Meningococcal Disease (blue)* [5].**

| **Age group**  **(years)** | **Case:carrier ratio** |
| --- | --- |
| <1  1-4  5-9  10-14  15-19  20-24  25-44  45-64  65+ | 0.004371  0.001098  0.000200  0.000080  0.000206  0.000079  0.000056  0.000304  0.002102 |

***Supplementary Table 2: Age-specific ACWY case:carrier ratio used in the model. This ratio was derived from modelled carriage incidence and observed IMD incidence, following pre-vaccination model fitting to carriage and case data in Supplementary Figures 1-2* [3][4][5]. *Due to data sharing constraints, values shown represent an average across broad age groups.***

Supplementary Figure 3 shows sensitivity analysis on the parameter for vaccine efficacy against carriage (κ). Model fitting suggested a suitable value of 41% (κ = 0.41). We have also depicted model behaviour with κ = 0.36, as observed by Read et al. [6]. We observed only minor differences in total carriage prevalence, even when running the model on a long timeframe.


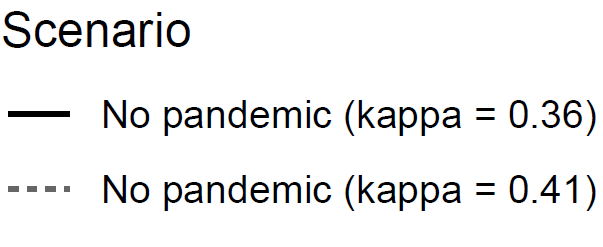

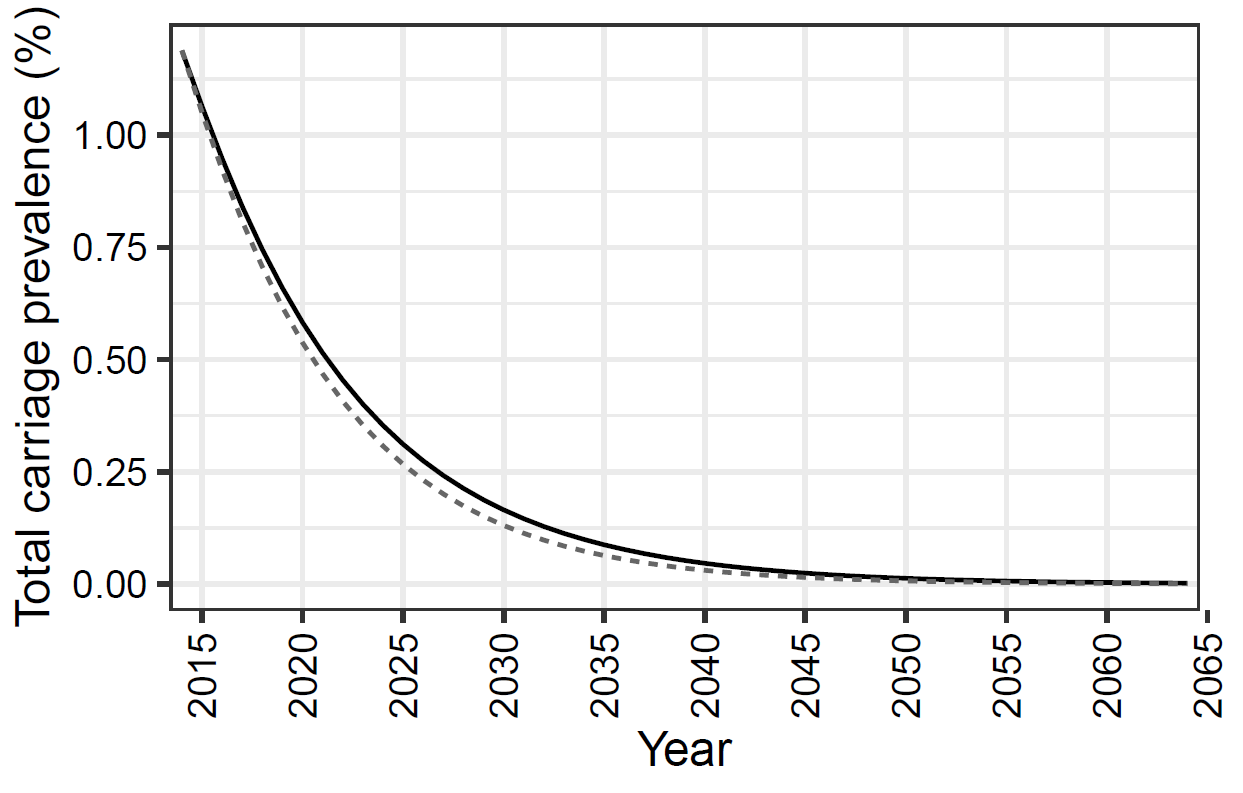


***Supplementary Figure 3: Exploring the parameter value choice for vaccine efficacy against carriage. We depict model runs with κ = 0.36 (Read et al.* [6]*; shown in black) and κ = 0.41 (base assumption; shown in grey).***

Supplementary Figure 4 shows sensitivity analysis on the parameter for reduced vaccine uptake (during the pandemic timeframe). We explored a larger reduced uptake of 50% and compare to the data-informed value of 34%. We observed only minor differences in carriage prevalence.

***
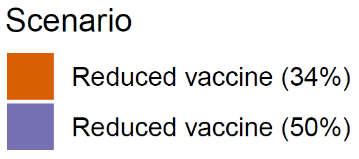

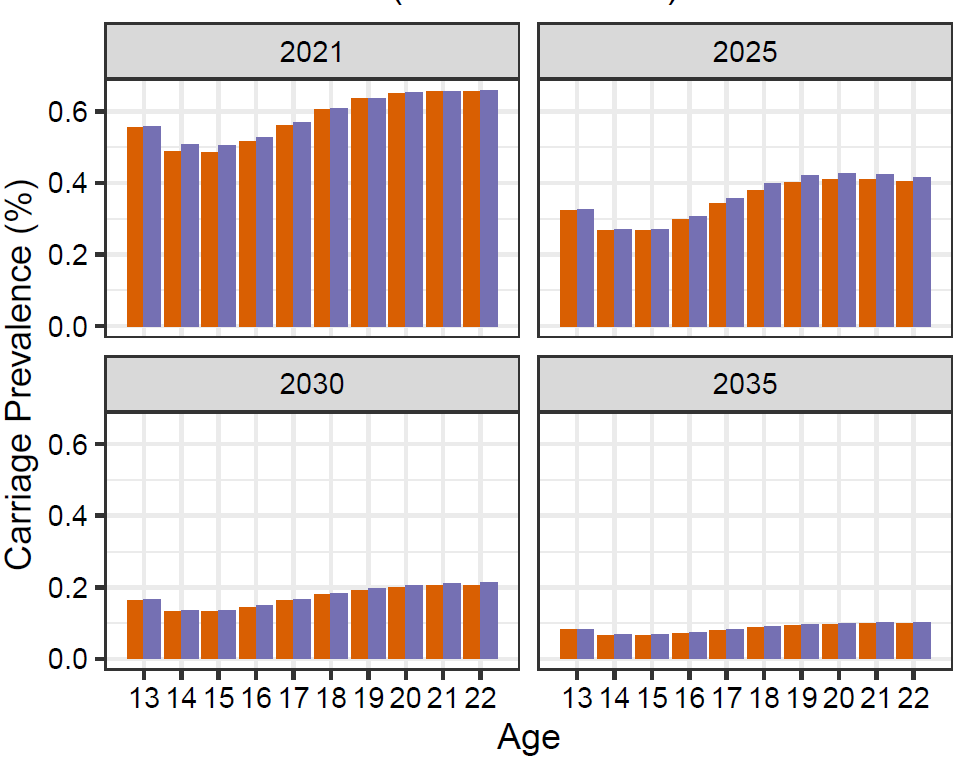
***

***Supplementary Figure 4: Exploring the parameter value choice for reduced vaccine uptake. We ran the model with a 50% reduction in vaccine uptake during the pandemic timeframe (purple). This was compared to the data-informed value of 34% (orange). These scenarios were run with the assumption of no social distancing.***

Supplementary Figures 5-6 depict sensitivity analysis on the parameter for pandemic social distancing. Instead of 60% and 75% reduction in daily social contacts for periods of school openings and closures, we modelled a 40% and 50% reduction respectively in Supplementary Figure 5. An immediate but long-lasting reduction in carriage was observed in both cases.

In Supplementary Figure 6, we used an age-stratified reduction in daily social contacts, informed by Gimma et al. [7]. For periods of school closure (April, May, June 2020 and January, February, March 2021), Gimma et al. observed an approximate 54% reduction in mean daily social contacts among 0-4 year olds, 74% reduction for 5-17 year olds, 77% reduction for 18-59 year olds, and 78% reduction for 60+ year olds, when taking an average of the two periods of school closure used in our model and comparing to the POLYMOD pre-pandemic baselines for each age group. Reduction in social mixing varied more by age group for the period July to December 2020 when pandemic restrictions were less stringent: 38% reduction for 0-4 year olds, 48% reduction for 5-17 year olds, 68% for 18-59 year olds, and 70% for 60+ year olds [7]. When running the model with these values to capture age-specific pandemic social distancing, we observed similar outputs to the original age-independent ‘Pandemic’ scenario. Carriage prevalence was slightly underestimated in adolescents and slightly overestimated in young adults.

***
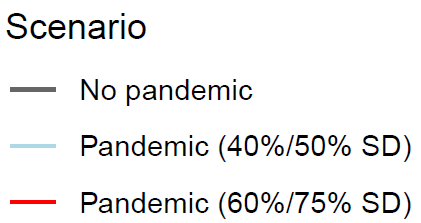

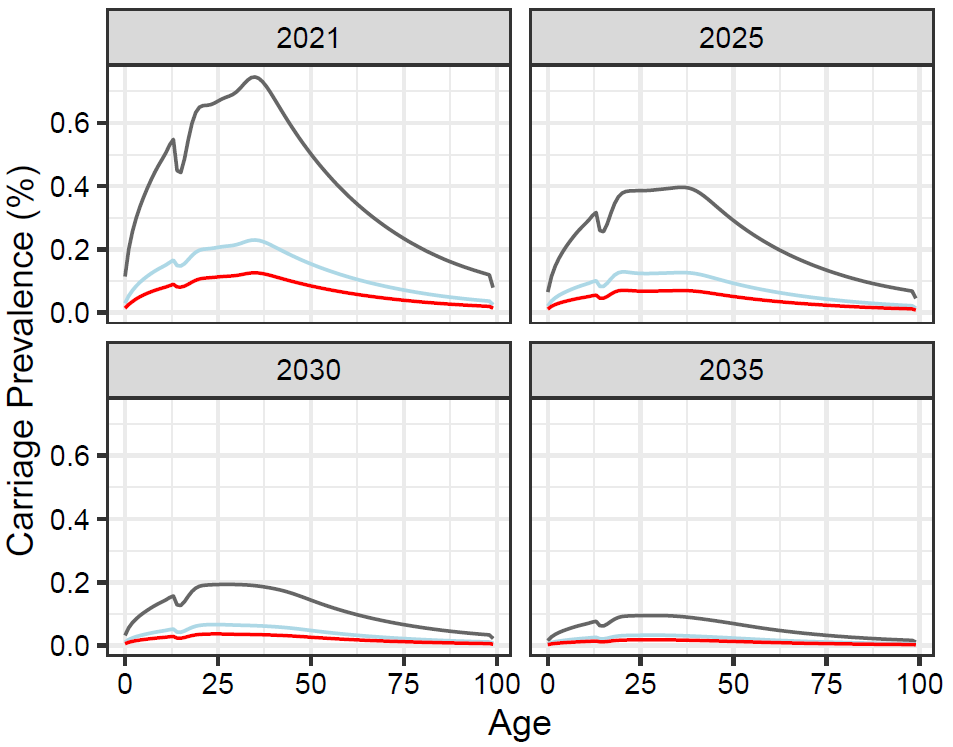
***

***Supplementary Figure 5: Exploring the parameter value choice for reduction in daily social contacts over the pandemic timeframe. A 40% and 50% reduction for periods of school openings and closures were modelled (light blue) and compared to the baseline assumption of 60% and 75% (red). The standard “no pandemic” scenario was also plotted for comparison (grey).***

***
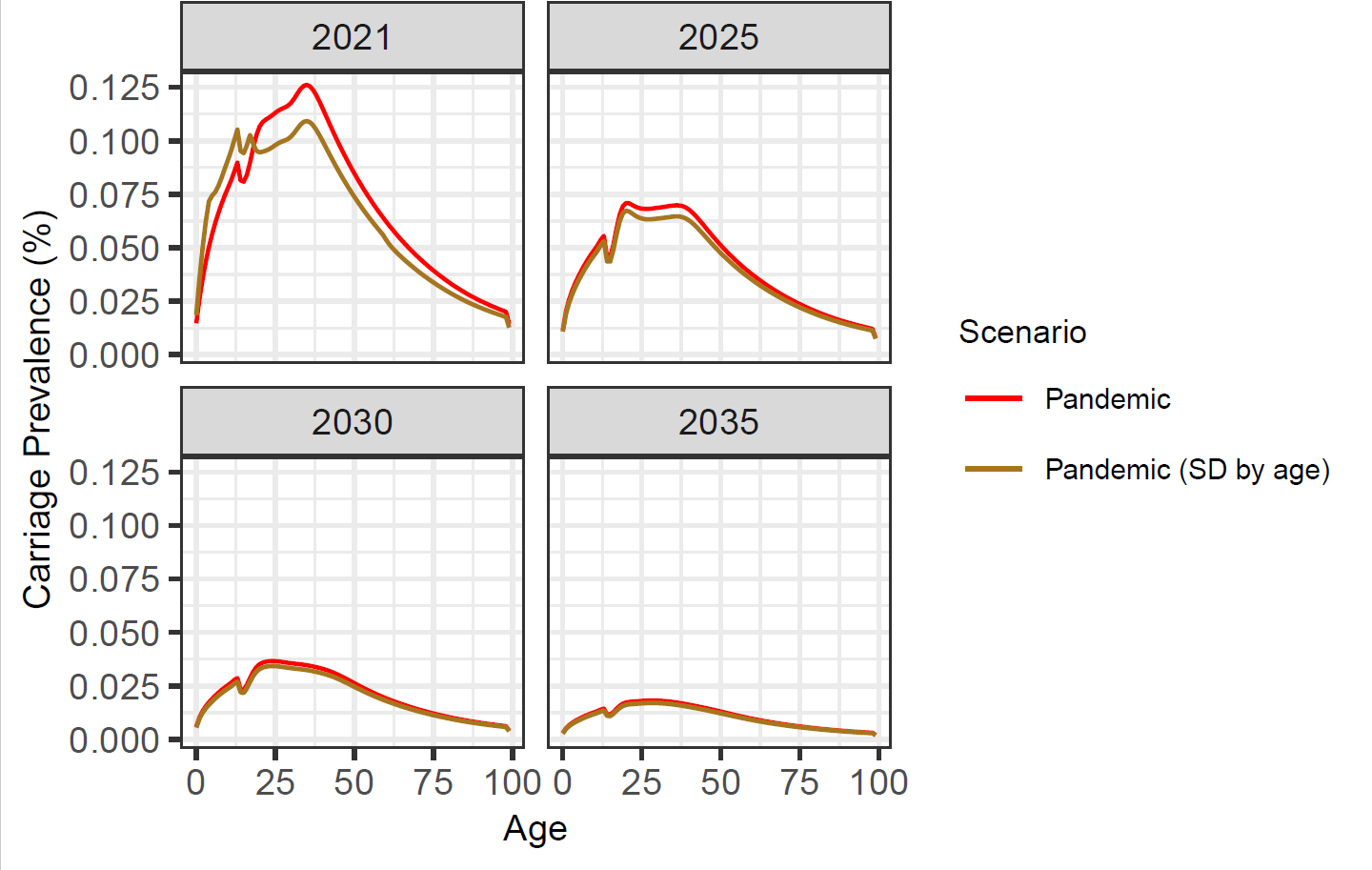
***

**a**

**
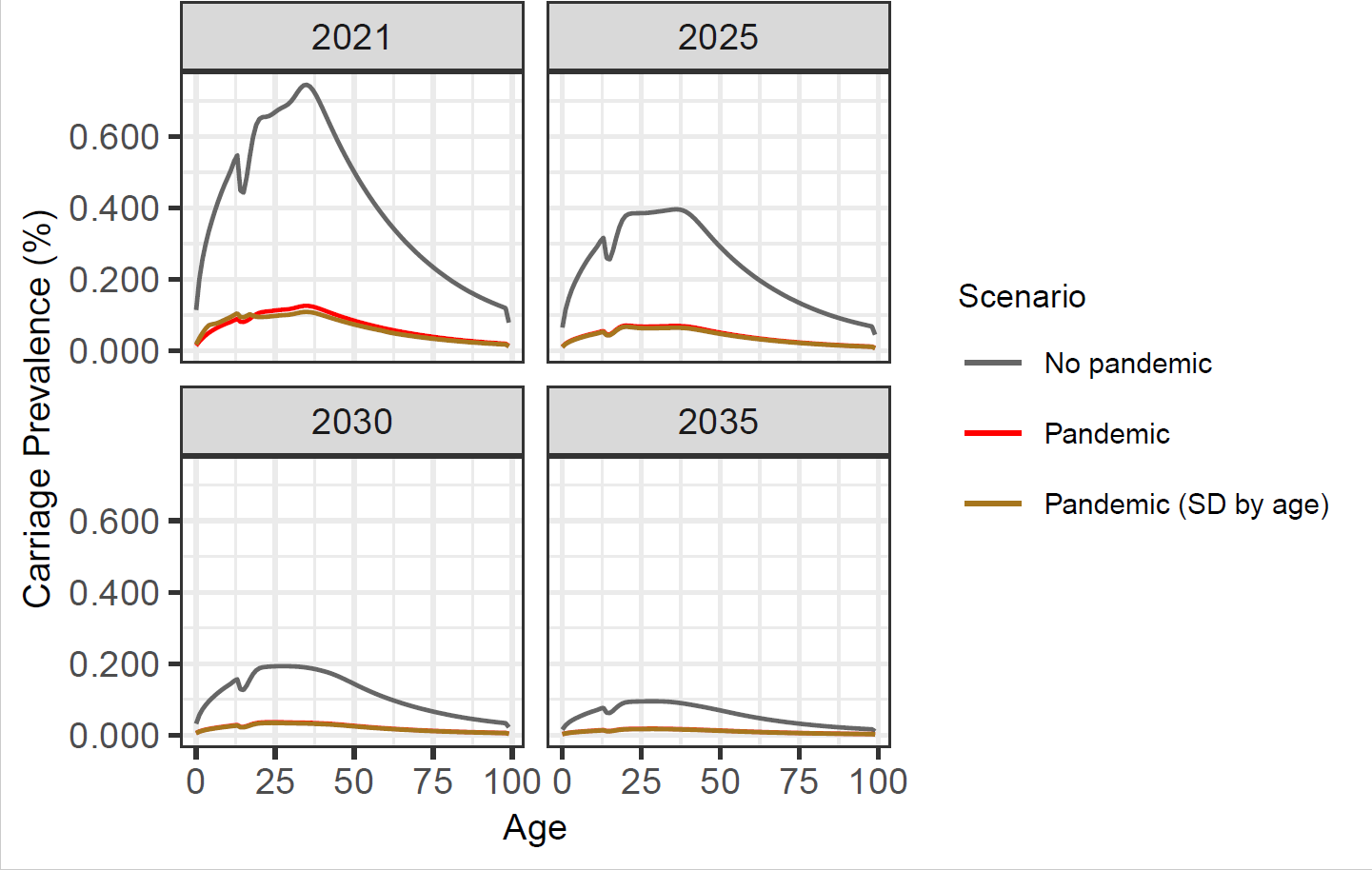
**

**b**

***Supplementary Figure 6: Exploring an age-stratified parameter for reduction in daily social contacts over the pandemic timeframe. We assumed 54%, 74%, 77%, and 78% reduction for periods of school openings and 38%, 48%, 68%, and 70% reduction for periods of school closures, in age groups 0-4y, 5-17y, 18-59y, and 60y+ respectively (brown). This was compared to the age-independent baseline assumption of 60% and 75% (red). The standard “no pandemic” scenario is also plotted for comparison in b (grey).***

Results for non-vaccine-preventable strains were not presented in the main text as this study was primarily focused on the changing prevalence of vaccine-preventable strains. However, individuals who contracted non-vaccine-preventable strains were still included in the model, and experienced reductions in pandemic social mixing in the same way as susceptible and ACWY-infected individuals. This is shown in Supplementary Figure 7. Note that a constant force of infection was used for non-vaccine-preventable strains.


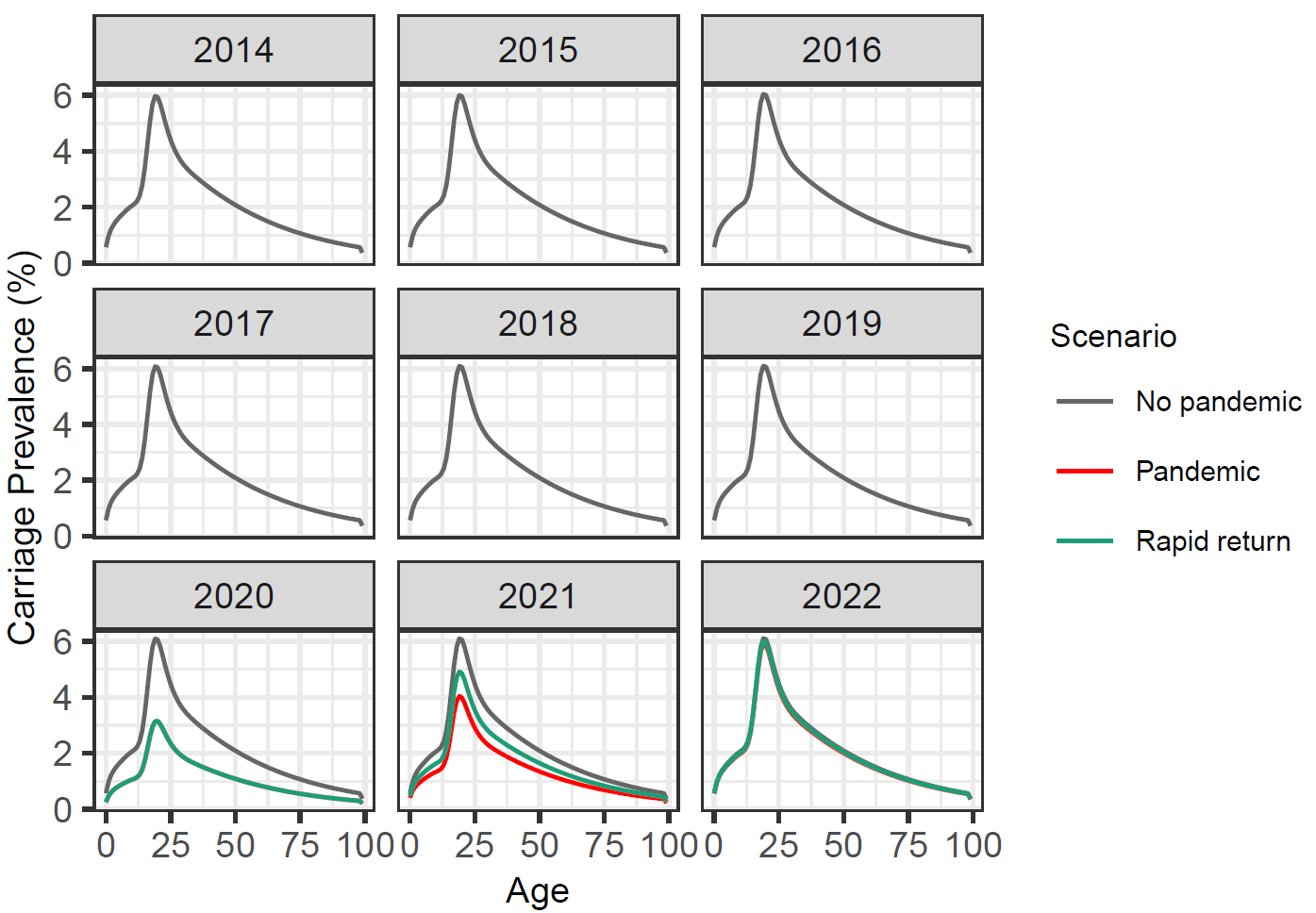


***Supplementary Figure 7: Behaviour of non-ACWY strains in the model.***

**References:**

1. **Christensen H, *et al.*** (2014) Re-evaluating cost effectiveness of universal meningitis vaccination (Bexsero) in England: modelling study. *BMJ*, **349**. <https://doi.org/10.1136/bmj.g5725>
2. **Christensen H, *et al.*** (2013) Introducing vaccination against serogroup B meningococcal disease: An economic and mathematical modelling study of potential impact. *Vaccine,* **31**. <https://doi.org/10.1016/j.vaccine.2013.03.034>
3. **Christensen H, *et al.*** (2010) Meningococcal carriage by age: a systematic review and meta-analysis. *The Lancet Infectious Diseases*, **10**. <https://doi.org/10.1016/S1473-3099(10)70251-6>
4. **MacLennan JM, *et al.*** (2021) Meningococcal carriage in periods of high and low invasive meningococcal disease incidence in the UK: comparison of UKMenCar1–4 cross-sectional survey results. *The Lancet Infectious Diseases*, **21**. <https://doi.org/10.1016/S1473-3099(20)30842-2>
5. **Public Health England**. (2015) *Invasive meningococcal disease (laboratory reports in England): 2014/2015 annual data by epidemiological year*. Retrieved from <https://www.gov.uk/government/publications/meningococcal-disease-laboratory-confirmed-cases-in-england-and-wales>
6. **Read RC, *et al.*** (2014) Effect of a quadrivalent meningococcal ACWY glycoconjugate or a serogroup B meningococcal vaccine on meningococcal carriage: an observer-blind, phase 3 randomised clinical trial. *Lancet*, **384**. <https://doi.org/10.1016/S0140-6736(14)60842-4>
7. **Gimma A, *et al.*** (2022) Changes in social contacts in England during the COVID-19 pandemic between March 2020 and March 2021 as measured by the CoMix study: A repeated cross-sectional study. *PLoS Medicine,* **19**.
